# Supplementary material for: Structured flexibility in recurrent neural networks via neuromodulation
Source: bioRxiv. 2024 Jul 26:2024.07.26.605315. Preprint. [Version 1] doi: 10.1101/2024.07.26.605315 (PMC11291173; doi:10.1101/2024.07.26.605315)
Supplement: Supplement 1 [file NIHPP2024.07.26.605315v1-supplement-1.pdf]

bioRxiv preprint doi: https://doi.org/10.1101/2024.07.26.605315; this version posted July 26, 2024. The copyright holder for this preprint (which was not certified by peer review) is the author/funder, who has granted bioRxiv a license to display the preprint in perpetuity. It is made available under aCC-BY 4.0 International license.

Supplemental figures

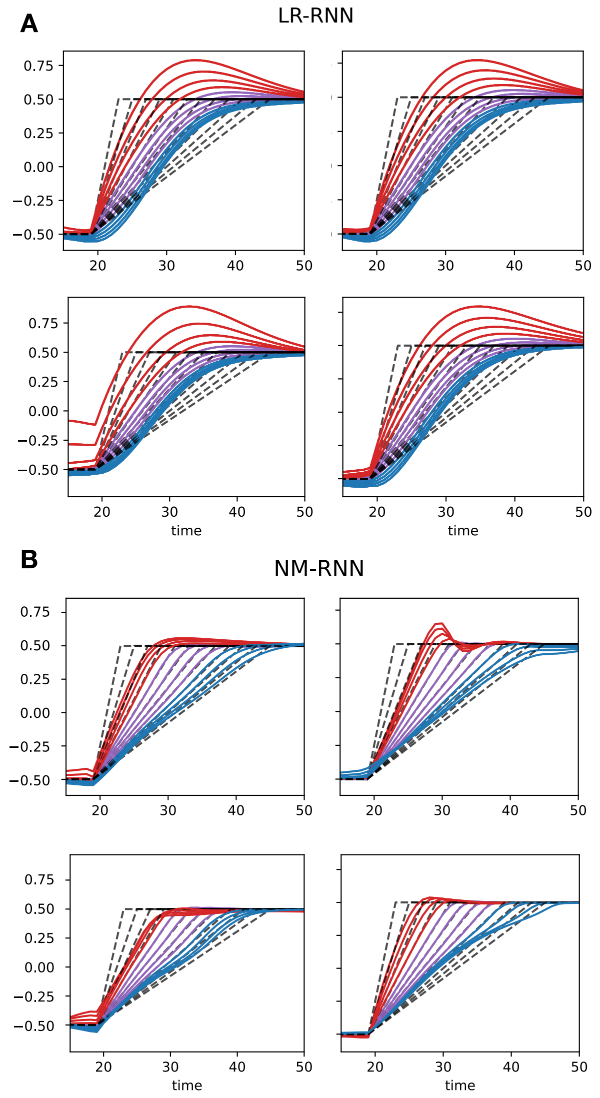

Figure S1: Example output comparison plots for the measure-wait-go task, as in fig. 3D in the main text, for four additional trained (**A**) low-rank RNNs and (**B**) NM-RNNs. Colors indicate extrapolated/trained intervals as in the main text.

bioRxiv preprint doi: https://doi.org/10.1101/2024.07.26.605315; this version posted July 26, 2024. The copyright holder for this preprint (which was not certified by peer review) is the author/funder, who has granted bioRxiv a license to display the preprint in perpetuity. It is made available under aCC-BY 4.0 International license.

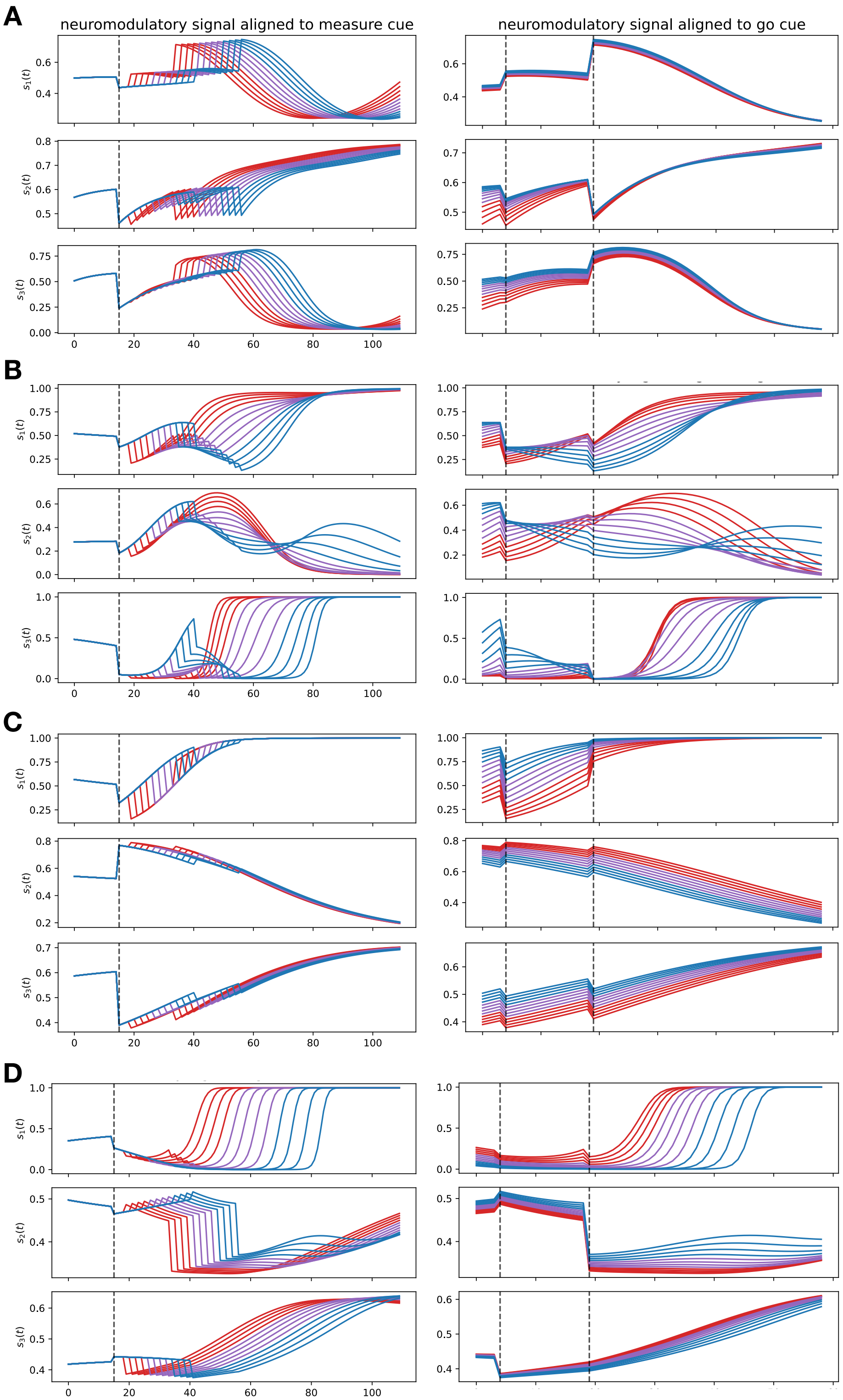

Figure S2: Example neuromodulatory signal plots for the measure-wait-go task, as in fig. 3E in the main text, for four additional trained NM-RNNs (same networks as shown in fig. S1B). Colors indicate extrapolated/trained intervals as in the main text.

bioRxiv preprint doi: https://doi.org/10.1101/2024.07.26.605315; this version posted July 26, 2024. The copyright holder for this preprint (which was not certified by peer review) is the author/funder, who has granted bioRxiv a license to display the preprint in perpetuity. It is made available under aCC-BY 4.0 International license.

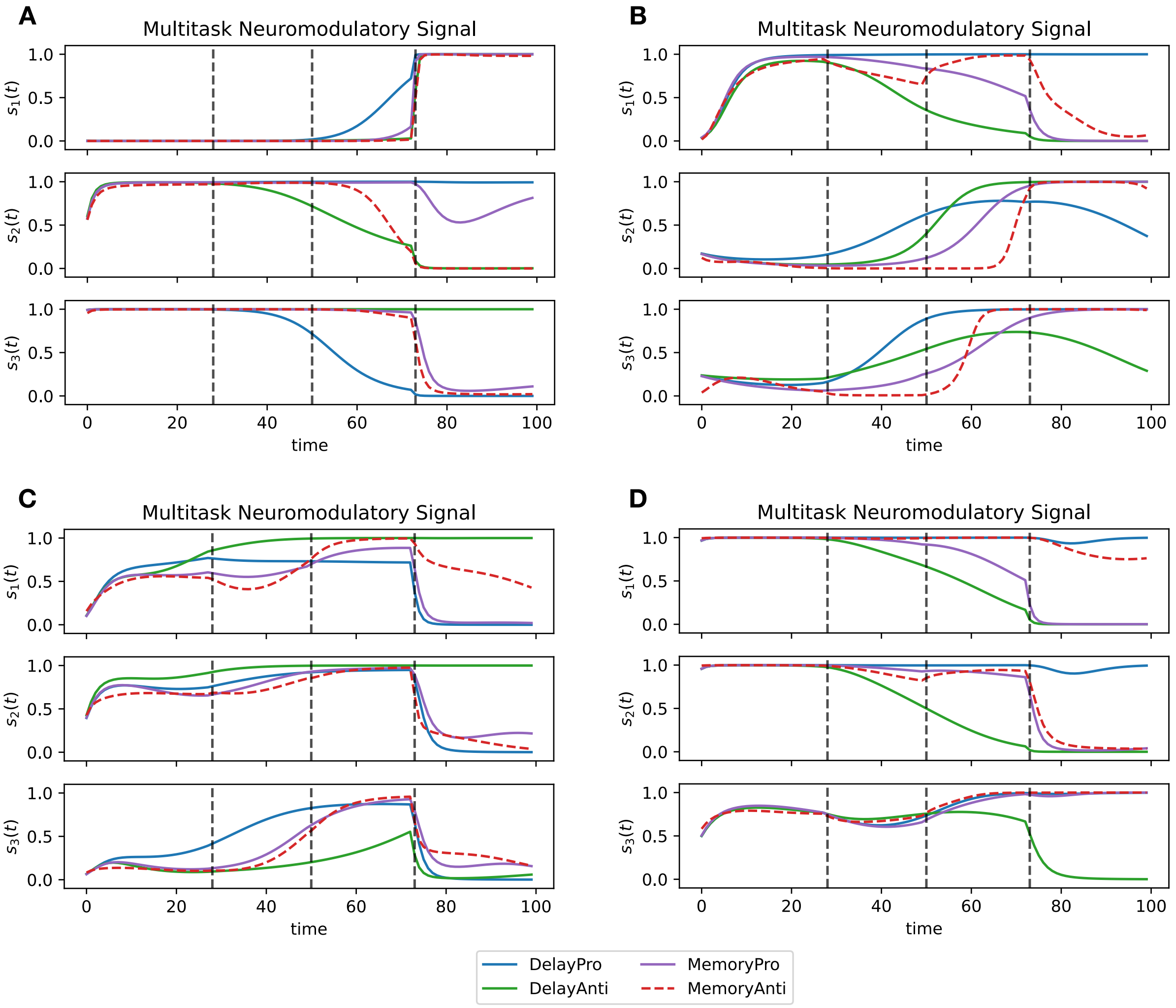

Figure S3: Example neuromodulatory signal plots for the multitask setting, as in fig. 4C in the main text, for four additional trained networks.

bioRxiv preprint doi: https://doi.org/10.1101/2024.07.26.605315; this version posted July 26, 2024. The copyright holder for this preprint (which was not certified by peer review) is the author/funder, who has granted bioRxiv a license to display the preprint in perpetuity. It is made available under aCC-BY 4.0 International license.

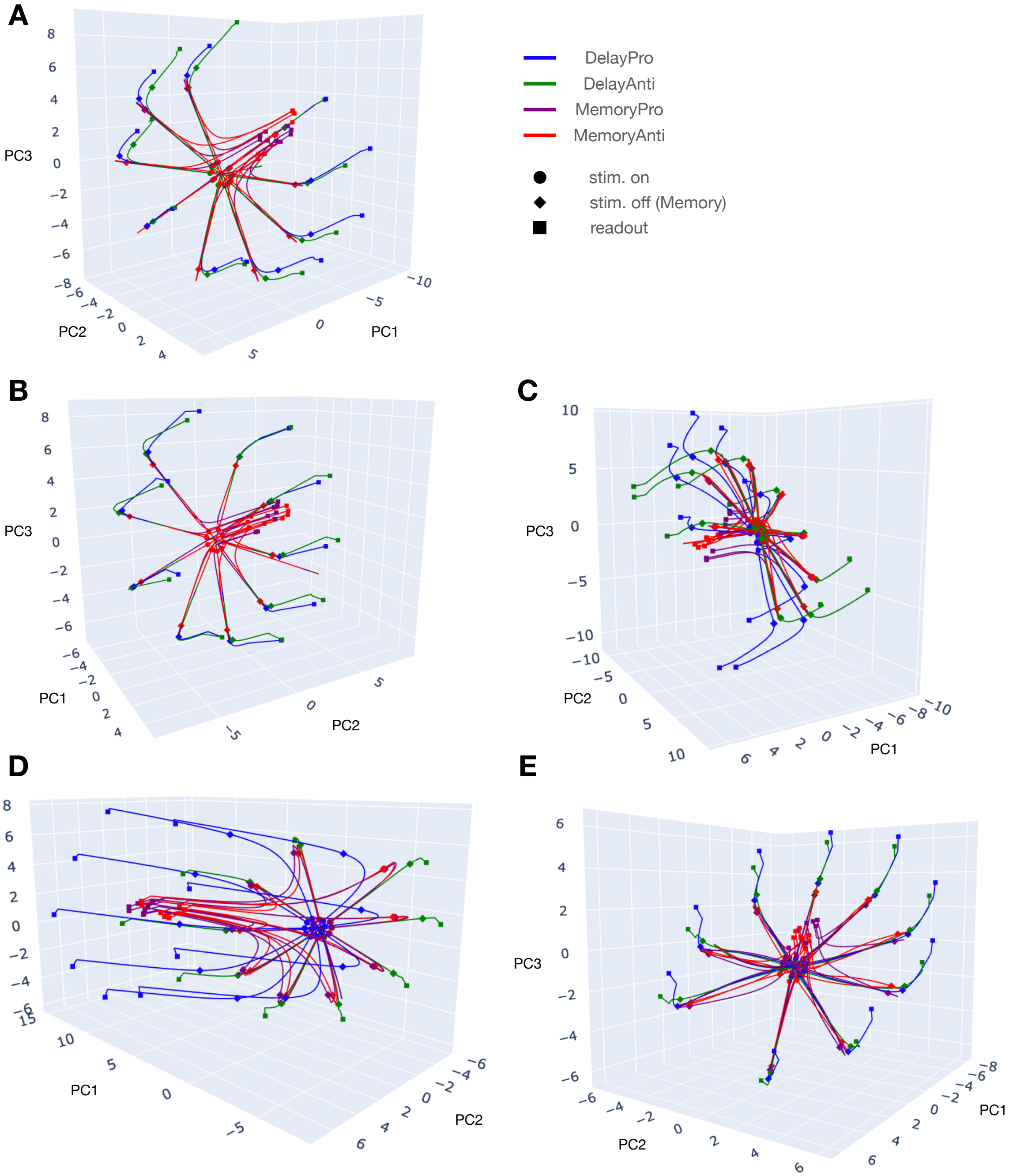

Figure S4: First three PCs of neural activity in multitask setting, plotted until readout period (for ease of visualization). **A.** Network visualized in main text, **B-E.** Four additional networks.

bioRxiv preprint doi: https://doi.org/10.1101/2024.07.26.605315; this version posted July 26, 2024. The copyright holder for this preprint (which was not certified by peer review) is the author/funder, who has granted bioRxiv a license to display the preprint in perpetuity. It is made available under aCC-BY 4.0 International license.

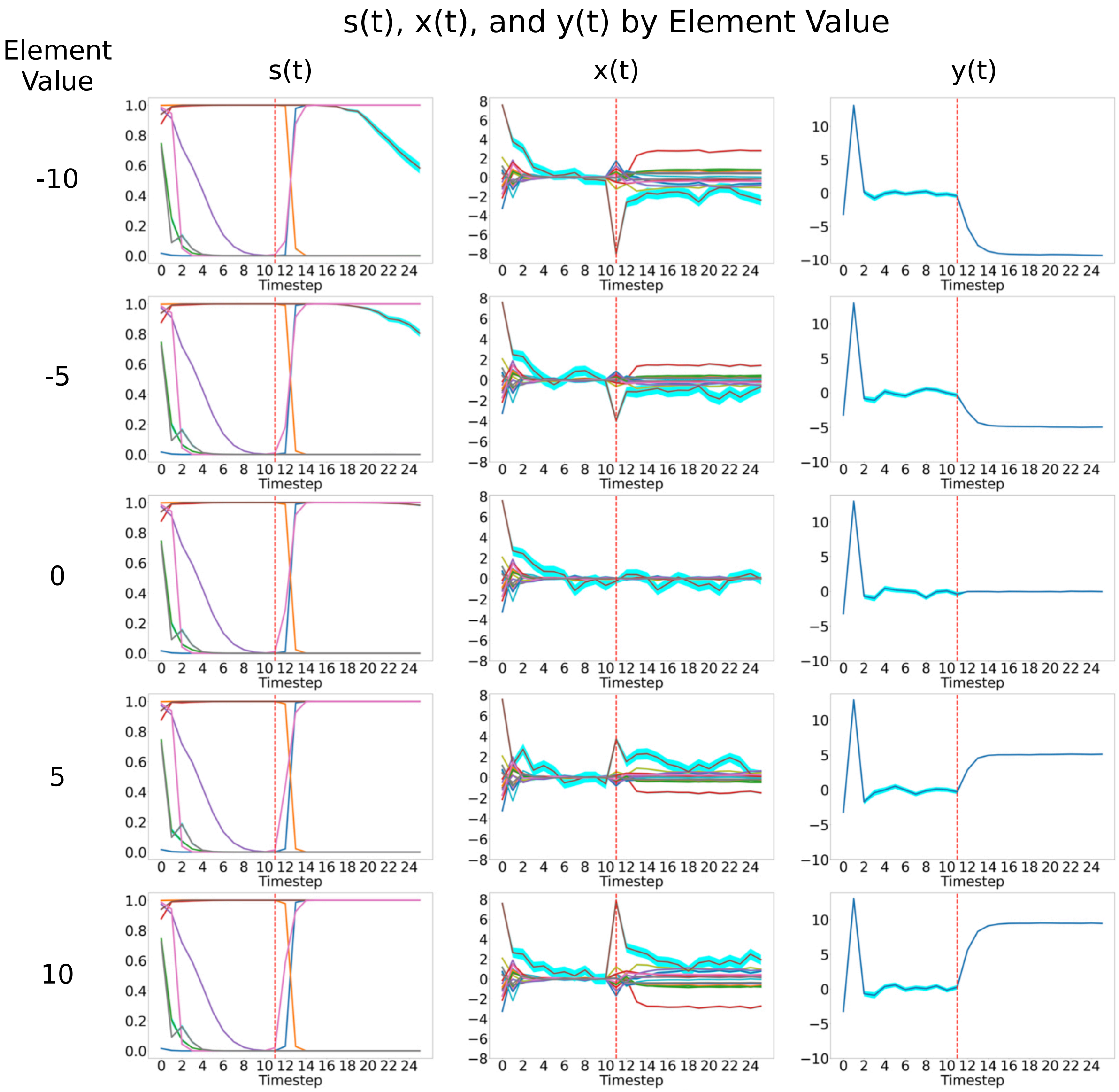

Figure S5: Sample internal states of an NM-RNN ($M = 5$, $N = 18$, $R = 8$) trained on the Element Finder Task, shown for 5 different element values (-10, -5, 0, 5, and 10). Each plot shows how all of the components of one of the vectors $s(t)$ (left), $x(t)$ (middle), $y(t)$ (right) vary through time. The query index is fixed to be 10, as indicated by the red dashed line in each plot. Each line shown is averaged over 100 independent runs of the model (standard error shown in cyan).

bioRxiv preprint doi: https://doi.org/10.1101/2024.07.26.605315; this version posted July 26, 2024. The copyright holder for this preprint (which was not certified by peer review) is the author/funder, who has granted bioRxiv a license to display the preprint in perpetuity. It is made available under aCC-BY 4.0 International license.

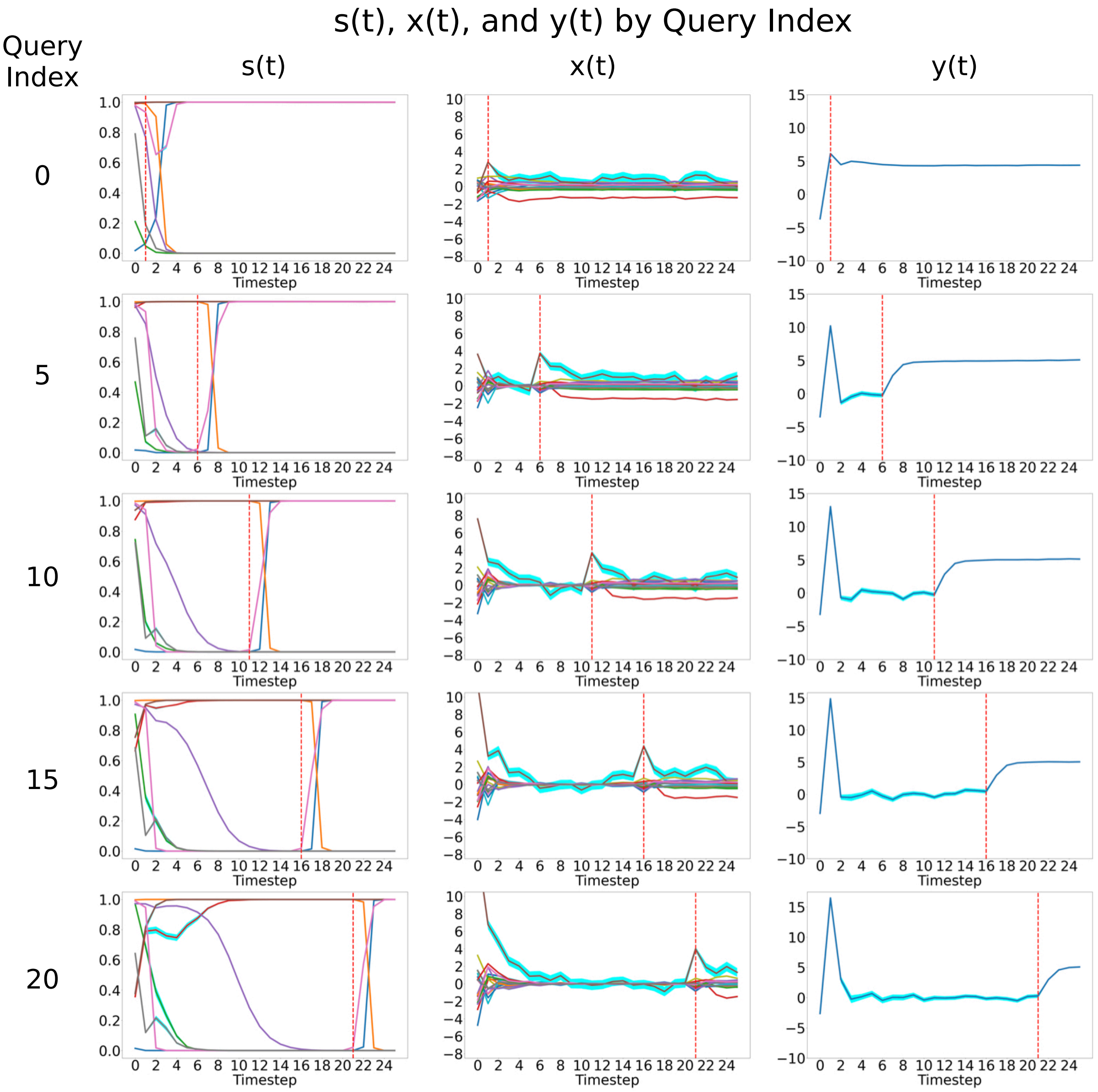

Figure S6: Sample internal states of an NM-RNN ($M = 5$, $N = 18$, $R = 8$) trained on the Element Finder Task, shown for 5 different query indices (0, 5, 10, 15, and 20), while fixing the target element value to be 5 in each case. Each plot shows how all of the components of one of the vectors $s(t)$ (left), $x(t)$ (middle), $y(t)$ (right) vary through time. In each plot, the onset of the query index is indicated by the red dashed line. Each line shown is averaged over 100 independent runs of the model (standard error shown in cyan).
